# Venom system variation and the division of labor in the colonial hydrozoan *Hydractinia symbiolongicarpus*

**DOI:** 10.1016/j.toxcx.2022.100113

**Published:** 2022-03-04

**Authors:** Anna M.L. Klompen, Steven M. Sanders, Paulyn Cartwright

**Affiliations:** a Department of Ecology and Evolutionary Biology, University of Kansas, Lawrence, KS, USA; b Department of Surgery, Thomas E. Starzl Transplantation Institute, University of Pittsburgh, Pittsburgh, PA, USA; c Pittsburgh Center for Evolutionary Biology and Medicine, University of Pittsburgh, Pittsburgh, PA, USA

**Keywords:** Cnidaria, *Hydractinia*, Nematogenesis, Venom modulation, Transgenesis, Transcriptomics

## Abstract

Cnidarians (jellyfish, hydroids, sea anemones, and corals) possess a unique method for venom production, maintenance, and deployment through a decentralized system composed of different types of venom-filled stinging structures called nematocysts. In many species, nematocyst types are distributed heterogeneously across functionally distinct tissues. This has led to a prediction that different nematocyst types contain specific venom components. The colonial hydrozoan, *Hydractinia symbiolongicarpus,* is an ideal system to study the functional distribution of nematocyst types and their venoms, given that they display a division of labor through functionally distinct polyps within the colony. Here, we characterized the composition and distribution of nematocysts (cnidome) in the different polyp types and show that the feeding polyp (gastrozooid) has a distinct cnidome compared to the reproductive (gonozooid) and predatory polyp (dactylozooid). We generated a nematocyst-specific reporter line to track nematocyst development (nematogenesis) in *H. symbiolongicarpus*, and were able to confirm that nematogenesis primarily occurs in the mid-region of the gastrozooid and throughout stolons (tubes of epithelia that connect the polyps in the colony). This reporter line enabled us to isolate a nematocyst-specific lineage of cells for *de novo* transcriptome assembly, annotate venom-like genes (VLGs) and determine differential expression (DE) across polyp types. We show that a majority of VLGs are upregulated in gastrozooids, consistent with it being the primary site of active nematogenesis. However, despite gastrozooids producing more nematocysts, we found a number of VLGs significantly upregulated in dactylozooids, suggesting that these VLGs may be important for prey-capture. Our transgenic *Hydractinia* reporter line provides an opportunity to explore the complex interplay between venom composition, nematocyst diversity, and ecological partitioning in a colonial hydrozoan that displays a division of labor.

## Highlights

• Functionally specific polyp types in *Hydractinia symbiolongicarpus* have distinct cnidomes.
• We present a nematocyst-targeted transgenic line for *H. symbiolongicarpus,* showcasing active areas of nematogenesis.
• 105 venom-like genes (VLGs) were annotated from an assembled nematocyst-enriched transcriptome.
• Several VLGs were significantly upregulated in feeding polyps, consistent with being a site of active nematogenesis.
• Differential expression analysis suggests that different polyp types express distinct combinations of VLGs.

## Graphical abstract

**Figure:**
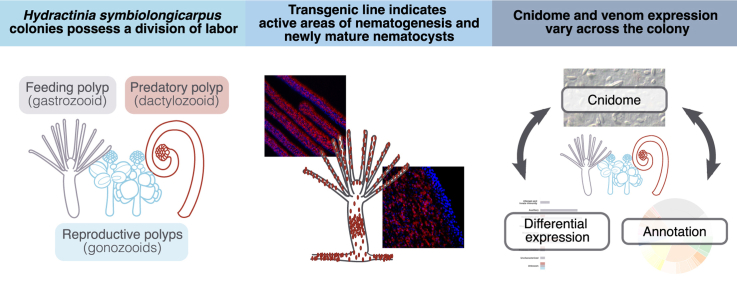

## 1 Introduction

A fundamental question in the evolution of venomous animals is how species manage the high metabolic cost of venom production and the utility of venom in multiple ecological interactions (i.e. venom metering; Morgenstern and King, 2013). Several phylogenetically-distant species have managed this tradeoff in such a way to simultaneously produce, maintain, and in some cases independently inject venom cocktails specific to the ecological context, including predatory and defensive venoms in cone snails (Dutertre et al., 2014; Abalde et al., 2021) and the assassin bug *Pristhesancus plagipennis* (Walker et al., 2018). Various strategies for modulating venom output appear to have emerged across the tree of life (Schendel et al., 2019).

Unlike other venomous animals, the Phylum Cnidaria (jellyfish, hydroids, sea anemones, and corals) has a decentralized venom delivery system composed of nematocysts, which are complex secretory organelles contained within cells that are distributed throughout the organism (Weill, 1934; Fautin et al., 2009). This decentralized system means the ecological utility of specific venom components and variation in venom expression can be analyzed using functionally specialized tissues and life stages (Ashwood et al., 2020). For example, the model sea anemone *Nematostella vectensis* Stephenson, 1935 has been a recent focus for studying venom modulation, including across the life history of this species (Columbus-Shenkar et al., 2018) and after exposure to abiotic stressors (Sachkova et al., 2020). Several studies in other sea anemones using RNA-seq show venom expression varies across distinct tissue types (Macrander et al., 2015, 2016; Surm et al., 2019; Ashwood et al., 2021a, 2021b). Although a robust pattern of toxin variation has been demonstrated in sea anemones (anthozoans), comparatively little has been explored in other cnidarians, including jellyfish and their relatives (medusozoans), despite their high developmental and ecological diversity **(**Boero et al., 2005). The few venom ecology studies outside of sea anemones have primarily focused on the freshwater model *Hydra* (reviewed in Sher et al., 2009; Rachamim et al., 2012) and medically-relevant species, such as box jellyfish (e.g. Underwood and Seymour 2007; McClounan et al., 2012). Additionally, a recent study in siphonophores shows that differential expression of putative toxins occurs across functionally specific tissues (Munro et al., 2021).

Cnidarians ability to regulate specific venom repertoires has been attributed to the distribution of different nematocyst types present in functionally different tissues. Nematocysts are ubiquitous across cnidarian species with ∼30 morphologically distinct types (Weill, 1934; Östman 2000; Fautin, 2009). The type and distribution across a species is a taxonomically-relevant characteristic called the cnidome (Weill, 1934), and many species typically contain 2-4 types across their ontogeny that are parsed out between different tissues or life stages (Fautin, 2009). Nematogenesis (development of nematocysts) has a characteristic set of stages, including capsule production followed by thread production, inversion of the thread into the capsule, and hardening and closing of the capsule (Holstein, 1981). The process is localized to a designated region and only mature or nearly mature nematocysts migrate to the specific tissue where they will be deployed (Kass-Simon and Scappaticci, 2002). It has been a long-standing question in cnidarian biology whether different nematocyst types contain different venom profiles, with evidence both for (e.g. McClounan et al., 2012; Columbus-Shenkar et al., 2018) and against (e.g. Brinkman et al., 2015; Doonan et al., 2019). This is further complicated by the lack of knowledge on when and where venoms are produced (see Lewis Ames and Macrander, 2016) and when during nematogenesis these toxins are packaged, although toxin expression appears to be expressed early and stops after maturation (Sunagar et al., 2018). Thus, the relationship between regulation of nematocyst type, venom expression, and the differential utilization of venoms is interconnected with nematogenesis.

**Fig. 1:**
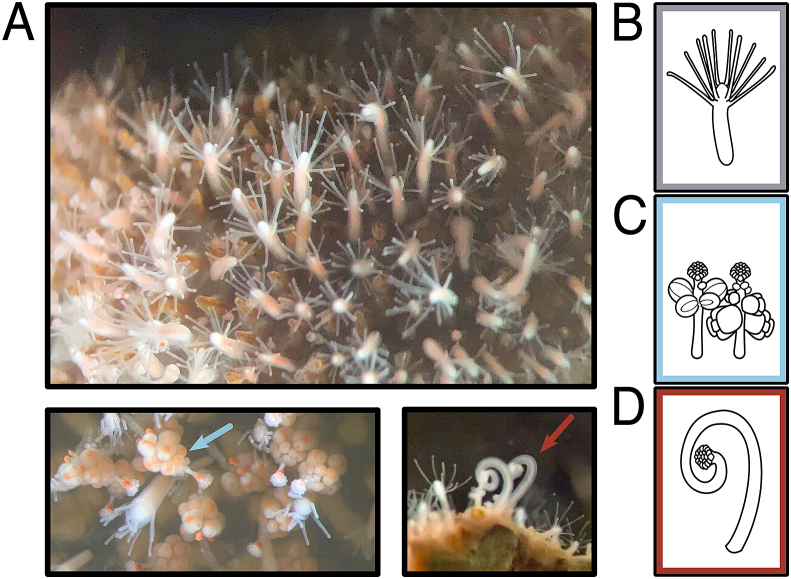
**Polyp types of the colonial cnidarian *Hydractinia symbiolongicarpus.*** A) Photos of morphologically and functionally distinct polyp types of *Hydractinia.* Top panel shows primarily gastrozooids, or feeding polyps, on the surface of a hermit crab shell. Bottom panels show close-ups of female gonozooids, or reproductive polyps, to the left (blue arrow) and dactylozooids, or prey-capture polyps, to the right (red arrow). Note that dactylozooids only appear at the opening of the hermit crab shell (Burnett et al., 1967). Lines drawings are provided of each polyp type of focus in this study: B) gastrozooid, C) gonozooids, male (left) and female (right), and D) dactylozooid.

*Hydractinia symbiolongicarpus*Buss and Yund (1989) is a genetically tractable cnidarian model system with functionally-distinct tissues and nematocyst types, which makes it ideal for exploring the complex relationships between nematogenesis, nematocyst composition and distribution, venom expression, and the ecological context of venom use. Species of the genus *Hydractinia* have been historically studied as a model in stem cell and developmental biology (reviewed in Frank et al., 2001; Frank et al., 2020). The North American species *H. symbiolongicarpus* has become increasingly popular due to its amiability to functional genomic techniques such as transgenesis (DuBuc et al., 2020), short-hairpin gene downregulation (Quiroga-Artigas et al., 2020), and CRISPR-Cas9 knock-downs and knock-ins (DuBuc et al., 2020; Sanders et al., 2018). *H. symbiolongicarpus* colonies possess a highly specialized organization comprising morphologically- and functionally-distinct polyp types: feeding and digestive polyps, which are the only type to possess a mouth (gastrozooids), male or female reproductive polyps (gonozooids), and predatory polyps (dactylozooids) (Fig. 1). These polyps are interconnected by branching gastrovascular canals called stolons, which carry nutrients throughout the colony as well as i-cells (hydrozoan stem cells) and nematocyst precursors (Frank et al., 2001). This division of labor ensures that functional specificity can be studied using the same colony of *Hydractinia*, minimizing the impact of genotype on toxin expression*.* There is also evidence to suggest that different polyp types do express distinct genes (Sanders et al., 2014), including putative toxins (Sanders et al., 2014; Klompen et al., 2021).

In order to study the interplay between the cnidome, nematogenesis, venom variation, and ecological use, we take advantage of the inherent ecological partitioning of *H. symbiolongicarpus* colonies as well as the functional genomic approaches available. Here, we characterize the cnidome across the different polyp types, generate a nematocyst-specific reporter to isolate nematocyst-enriched tissue for transcriptomic analysis, and integrate these findings with observations on how venom expression and nematogenesis are partitioned within the colony.

## 2 Methods

### 2.1 Animal care

*Hydractinia symbiolongicarpus* colonies were maintained at the University of Kansas on glass microscope slides in artificial seawater (ASW) (Instant Ocean Reef Crystals, USA) between 28 and 30 ppt at room temperature (22–23 °C). Parental colonies (male 291–10; female 295–8) and progeny from this study were maintained in separate 5-gallon tanks. Animals were fed 2–3 times each week with 2–3 day old brine shrimp nauplii (*Artemia* sp.) or frozen mussel and given 10–20% water changes after each feeding.

Shells of the dwarf hermit crab (*Pagurus longicarpus* Say, 1817) with established *H. symbiolongicarpus* colonies were purchased from Marine Biological Laboratories (Woods Hole, MA, USA). Animals were maintained in a 15-gallon tank with ASW in the same conditions as the cultured *H. symbiolongicarpus* colonies. Crabs were provided frozen shrimp or mussels 2–3 times a week, followed by 2–3 day old brine shrimp for hydroid colonies. Water changes were conducted weekly, varying from 10 to 50% depending on water quality.

### 2.2 Nematocyst identification and counting

**Fig. 2:**
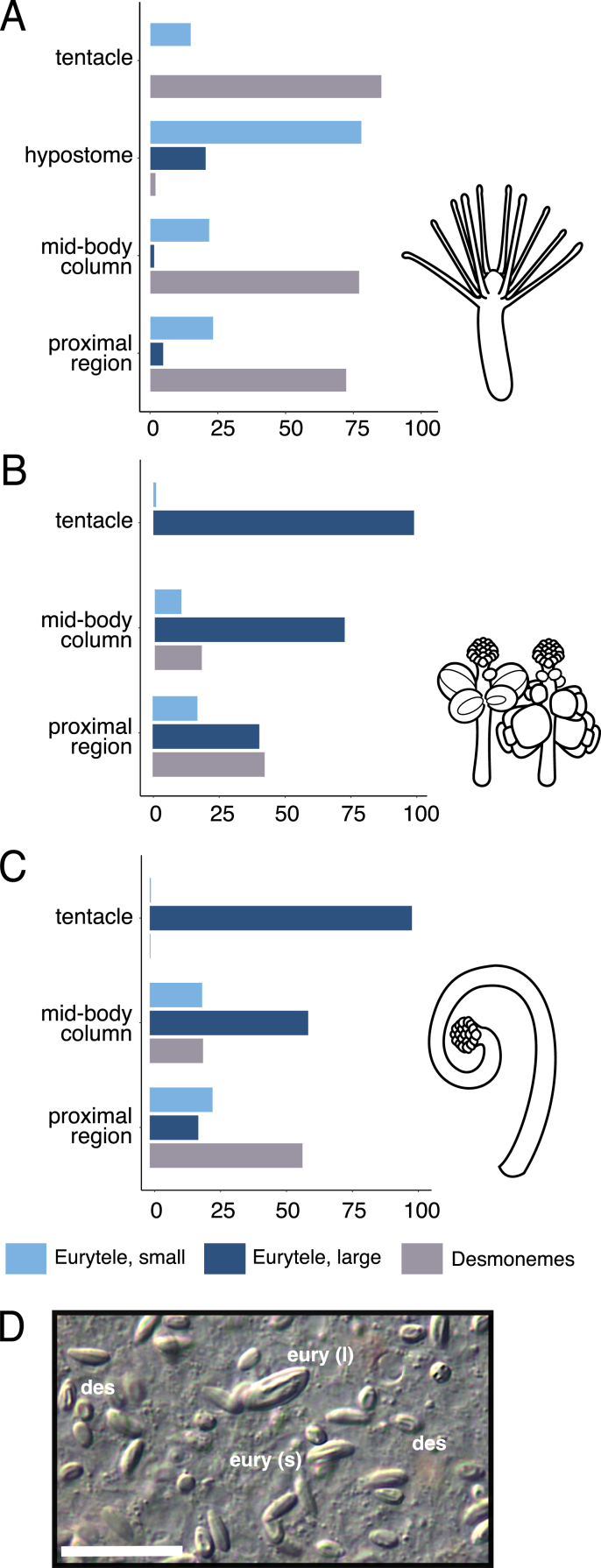
Cnidome distributions across each polyp type of *Hydractinia*. Proportion of nematocyst types across sections of each polyp type: gastrozooid (A), gonozooids (B), and dactylozooid (C). Percentages are based on total counts from 2 to 3 individual polyps each from three different colonies. Proximal region refers to the lower 25% of the body column. D) Undischarged large eurytele (l), small eurytele (s), and desmonemes (des) in the body column of gastrozooid. Scale bar = 20 μm.

Nematocyst classification was based on Östman (2000) and previous reports on *Hydractinia* spp. (Mills, 1976; Buss and Yund, 1989; Lange et al., 1989; Schuchert, 2008, 2014). Three different hermit crabs containing colonies with >50% coverage were randomly selected and three gastrozooids, dactylozooids, and at least two gonozooids (male or female) from each crab were used for cnidome characterization. Polyps were placed on glass slides and covered by coverslips containing small amounts of clay at the corners to ensure the integrity of each polyp. Images were taken using a Zeiss Axioscope 2 Plus microscope and Lumenera Infinity 3 camera with Infinity Analyze v10 software. The tentacles of each polyp, hypostome (in gastrozooids only), mid-body column, and the lower 25% of the polyp (proximal region) were imaged individually, and all nematocyst types that could be confidently assigned based on orientation were counted. Calculations and data visualization were conducted in R v4.1.2 (R Core Team, 2021) with RStudio v2021.9.0.351 (RStudio Team, 2021). Results are shown in Fig. 2. All figures were created using Inkscape v1.1.

### 2.3 Fertilization and development

Breeding colonies were kept on a 8/16 h light-dark cycle. After exposure to light, males and females were placed in separate containers with fresh ASW. Spawning was induced within 1–1.5 h, and water containing released eggs was combined with 10–20 ml of water containing released sperm with gentle stirring. After ∼10 min, the fertilized eggs were concentrated for microinjection, if applicable. Embryos were moved to glass petri dishes with fresh ASW to develop into planulae. After 72–96 hpf, metamorphosis was induced with a 4–6 h incubation in 56 mM CsCl. The planulae were placed on glass coverslips or petri dishes to settle, and primary polyps developed over the next 48 h. Primary polyps are initially fed 1–2 day old brine shrimp using a pipette until the animals were mature enough to capture prey unassisted.

### 2.4 DNA extraction

Tissues were prepared by scraping polyps from a 4-day starved male colony with a razor blade, transferring to a 1.7 ml centrifuge tube, and briefly spinning down to remove excess ASW. DNA was extracted using a DNeasy Blood and Tissue Kit (Qiagen Cat.69504) according to the manufacturers’ protocol. The resulting extract was eluted into nuclease-free water and stored at −20 °C until use.

### 2.5 Identification, cloning, and synthesis of *Ncol-1: mScarlet* plasmid

*Minicollagen-1* (*Ncol-1*) was identified by querying the sequence of *Ncol-1* from sister species *H. echinata* (NCBI Acc. BK008461.1) against a previously published *H. symbiolongicarpus* RNA-sequencing dataset (Sanders and Cartwright 2015) using *blastn* v2.8.1+. The resulting sequence was validated against a draft genome of *H. symbiolongicarpus* ((Hydractinia Genome Project Portal, 2021); https://research.nhgri.nih.gov/hydractinia/) to identify the full CDS (start and stop codons included). Primers were designed to capture a 1968 bp region upstream of the start codon and a 1429 bp region downstream of the stop codon. The two fragments were PCR-amplified using Phusion High-Fidelity DNA Polymerase (ThermoFisher Scientific, Cat. F530) and subsequently cloned using TOPO pCR2.1 Cloning Kit (ThermoFisher Scientific, Cat. K202020), screened, and verified via sequencing (GENEWIZ, USA).

**Fig. 3:**
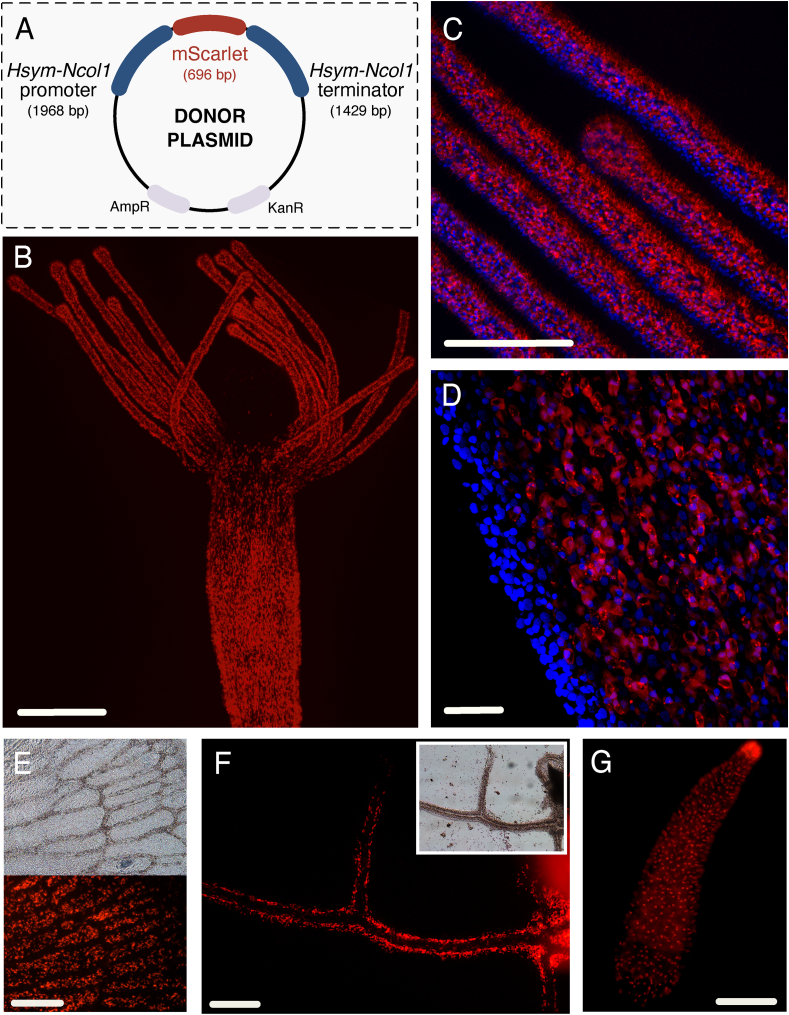
Establishment of a *Ncol-1::mScar* transgenic. A) Schematic of donor plasmid created and injected into *Hydractinia* embryos; full coding domain sequence of a codon-optimized mScarlet-I (DuBuc et al., 2020) is flanked by a putative promoter and terminator sequence of *Ncol-1* obtained from the genome of *Hydractinia*. B) Live confocal image of full view of gastrozooid; strong signal in the central body column and tentacle regions. C) Close-up of tentacles in fixed gastrozooid. D) Close-up of the edge of the body column in fixed gastrozooid. E) Close-up of stolonal mat in growing colony; bottom shows fluorescent signal coming from *Ncol-1*^+^ cells. F) Branching stolon from the central colony shows strong fluorescence; inset shows the same tissue using light microscopy. G) Transgenic planula 24 hpf showing widespread *Ncol-1*^+^ cells, consistent with nematoblasts and recently matured nematocysts. B-D) Imaged using Leica Laser Scanning Confocal Upright microscope; E-G) Imaged using Lumenera Infinity camera with Infinity Analyze v7 software. Blue = DAPI, red = mScarlet. Scale bar: 200 μm (B, E-G), 100 μm (C), and 20 μm (D). (For interpretation of the references to color in this figure legend, the reader is referred to the Web version of this article.)

Custom Gibson primers for the *Ncol-1* promoter and terminator fragments, as well as a previously published *Hydractinia* codon-optimized *mScarlet-I* plasmid (pUP730_Btub-mScarlet; DuBuc et al., 2020; Bindels et al., 2017), were designed using NEBuilder Assembly Tool v2.2.8. All three fragments were amplified using Q5 High-Fidelity 2X Master Mix (NEB, Cat. M0492) and assembled using Gibson Assembly Master Mix (NEB, Cat. E2611S) according to the recommended protocol. The resulting Gibson assembly reaction was cloned into TOPO pCR2.1, screened by restriction mapping, and sequenced to validate the assembly of *Ncol-1::mScarlet* plasmid (Fig. 3A; Suppl. File S1).

### 2.6 Microinjection, screening, and imaging

For microinjection, *Ncol-1::mScarlet* plasmid was extracted using a Plasmid Midi Kit (Qiagen, Cat. 12143), with an additional phenol/chloroform purification, isopropanol precipitation, and resuspension in Milli-Q H_2_O (Millipore Sigma, USA). This stock solution was diluted in Milli-Q H_2_O and stored at −20 °C until use. Approximately 10 min post-fertilization, embryos were injected with the purified plasmid at a concentration between 500 and 1000 ng/μl (Künzel et al., 2010). Non-injected animals in the same batch served as a developmental control and no delays were observed. Fluorescence could be seen in mosaic *Ncol-1::mScarlet* F0 animals 24 h post-injection, matching previous *in situ* expression patterns (Kanska and Frank, 2013). Healthy F0 mosaics were settled and only animals that maintained fluorescence after settlement were raised to maturity. One high-signal F0 female colony was crossed with the 291–10 wild-type male to establish stable F1 offspring. A robust F1 female, referred to as *Ncol-1::mScar*, was selected for use in all subsequent experiments given that the distribution of fluorescent markers was consistent with expected sites of nematogenesis and newly matured nematocysts (Fig. 3; Kanska and Frank, 2013).

To ensure that the signal was localized to the correct cell population, fluorescent confocal images of the transgenic colony were taken using a Leica Laser Scanning Confocal Upright Microscope at the University of Kansas Microscopy and Analytical Imaging Core. Animals were imaged live or fixed using 4% PFA for ∼1 h at RT, washed three times, and mounted with 90% glycerol/10% PBS with 0.001% DAPI.

### 2.7 Tissue dissociation and FACS

The female *Ncol-1::mScar* colony was starved for 3-days prior to sorting. Approximately 50 polyps (40 gastrozooids + 10 gonozooids) were removed from the colony using a razor blade and relaxed in menthol for 60 min prior to dissociation. Dactylozooids are difficult to culture in laboratory conditions (see Burnett et al., 1967) and thus not included in this dataset. Polyps were moved to a 1.7 ml tube and cleaned multiple times with calcium and magnesium-free artificial seawater (CMASW) with EGTA. Animals were dissociated in 400 μl of 1% pronase (Sigma-Aldrich, Cat. #10165921001) over 1.5 h at room temperature with constant rocking, as well as gentle mixing with a glass pipette every 15 min. Cells were filtered through a 70 μm Flowmii cell strainer (Millipore Sigma, Cat. BAH136800070) and pelleted at 300rcf for 5 min at 4 °C. The pellet was resuspended in ice-cold 0.1% BSA in CMASW. Prior to sorting, cells were filtered through a 40 μm cell strainer and DAPI (0.125 μg/ml) was added to ensue cells could be separated as “live” (low DAPI-staining) or “dead” (high DAPI-staining).

Fluorescence activated cell sorting (FACS) was conducted at the Flow Cytometry Core Laboratory at the KU Medical Center (KUMC) using a FACSAria IIIu (BD Biosciences, USA) cell sorter with a 100 μm nozzle and sheath pressure at 20 psi. To prevent potential osmotic stress, CMASW was used for the sheath, following DuBuc et al. (2020). The sorter was equipped with 405 and 633 nm excitation lasers in order to sort “live” mScarlet-positive samples and mScarlet-negative samples. Cells were sorted into 2 ml tubes coated with CMASW and afterwards stored on ice. Sorting was evaluated using FACSDiva v8.0 (BD Biosciences, USA) (Suppl. Fig. S1), and an aliquot of sorted cells were manually evaluated under a fluorescent microscope (Suppl. Fig. S2).

### 2.8 RNA isolation, sequencing, and transcriptome assembly

After pelleting cells to a final solution of 500 μl, RNA isolation, library construction, and sequencing of FACS cell samples was performed at the KUMC Genome Sequencing Facility on NovaSeq 6000+ V1.5 SBS platform. Read quality pre-assessment, filtering, and quality verification were implemented using *fastp* v0.20.1 (https://github.com/OpenGene/fastp) using base correction (-c), phread value cutoff of 30 (-q 30), length cutoff of 36 (-l 36), and sliding window from front to tail with a cutoff of phred quality 30 (-r --cut_right_mean_quality 30). The resulting reads were assembled *de novo* using Trinity v2.8.5 (Haas et al., 2013) (Suppl. Table S1). We validated the increased expression of *Ncol-1* in the *mScarlet*^*+*^ sample (herein referred to as “bright sample) compared to the negative samples (see Section 2.10; Suppl. Table S2). Raw sequencing data from this project have been deposited into the Sequence Read Archive at the National Center of Biotechnology Information (NCBI BioProject PRJNA809365).

### 2.9 Venom-like gene identification and annotation

**Fig. 4:**
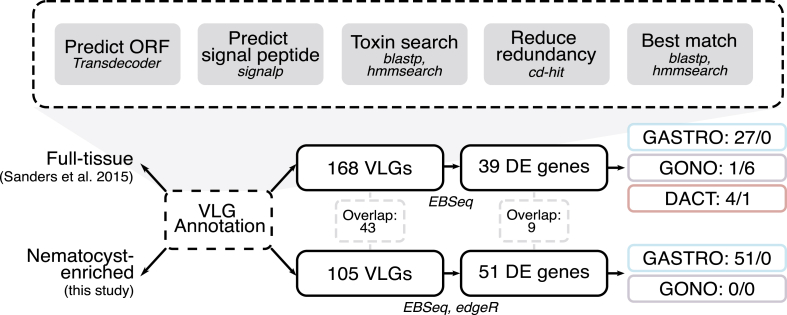
Schematic of bioinformatic pipeline for venom-like gene (VLG) annotation and DE in *Hydractinia*. For each polyp type (colored boxes), the left number represents the number of upregulated genes and the right number represents the downregulated genes.

Putative venom-like gene (VLGs) annotation was based on previously published pipelines (Klompen et al., 2020, 2021) (Fig. 4). Transcripts were translated into putative protein-coding regions using Transdecoder v5.5.0 and those with a signal peptide were filtered using SignalP v5.0b. An initial *blastp* v2.9.0+ search was conducted using the UniProtKB/Swiss-Prot Tox-Prot program (ToxProt; Jungo et al., 2012) and all NCBI proteins found using search terms “Cnidaria AND ((Toxin) OR (Venom))". Both databases were downloaded in July 2021. Additional searches were conducted against customized cnidarian-toxin gene family and domain-specific HMM models (modified from von Reumont et al., 2014; Verdes et al., 2018; Klompen et al., 2020, 2021) using *hmmsearch* (HMMER v3.3.2; http://hmmer.org/). All annotation searches were conducted using an E-value cutoff of 0.001. The resulting sequences were filtered for 98% identity (-c 0.98) using CD-HIT v4.8.1 (http://weizhong-lab.ucsd.edu/cd-hit/; Huang et al., 2010), then used as queries against the ToxProt, Swiss-Prot as well as the NCBI nr database (downloaded August 2021) using *blastp* and domain annotation against the Pfam database (downloaded August 2021; Mistry et al., 2021) using *hmmsearch*, all with an E-value cutoff of 0.001. The results were manually curated to determine putative VLGs. Uncharacterized indicates that a transcript had a best match to an uncharacterized protein from NCBI; unknown indicates that no annotation was given from any database. The same protocol as above was used to identify putative VLGs within an assembled transcriptome for mixed full polyp tissues from multiple *Hydractinia* colonies (TSA: GCHW00000000.1; Sanders and Cartwright, 2015).

### 2.10 Differential expression across polyp types

Reads from different polyp tissues (BioProject PRJNA237004; Sanders et al., 2014) were mapped to the nematocyst-enriched transcriptome and a full-tissue transcriptome using RSEM v1.3.1 (Li et al., 2011) under default settings, and the gene results were used for downstream analysis. To maintain consistency, raw reads for the full-tissue datasets were quality filtered using the same conditions as the reads in this study. Additionally, *mScarlet*^*+*^ “bright” and negative reads from this study were mapped to the nematocyst-enriched transcriptome to obtain TPM values. For the nematocyst-enriched transcriptome, differential expression analyses were conducted using EBSeq v1.34.0 (Leng et al., 2013) and edgeR v3.36.0 with the quasi-likelihood F-tests (Robinson et al., 2010) to compare the gastrozooid and gonozooids reads. Only genes found from both tests were considered differentially expressed. Only EBSeq was used to determine differential expression of VLGs between gastrozooids, gonozooids, and dactylozooids in the full-tissue dataset.

## 3 Results

### 3.1 Nematocyst distribution across polyp types in *Hydractinia*

Three morphologically distinct nematocyst types were identified in *Hydractinia*: desmonemes, small euryteles, and large euryteles, consistent with previous studies (Mills, 1976; Lange et al., 1989; Schuchert 2008, 2014) (Fig. 2). In addition to differences in size, small and large euryteles are identifiable based on slightly different capsule morphologies, including slight swelling of the capsule in large euryteles (Fig. 2D). Within the gastrozooid, adhesive desmonemes are the most common type found within the filiform tentacles at ∼75%, with the remaining ∼25% consisting of small euryteles evenly scattered across the tentacles (Fig. 2A). Smaller euryteles are the only nematocyst type found in the hypostome (region that contains the mouth) of gastrozooids. Gonozooids and dactylozooids contain >99% large euryteles in their short capitate tentacles (Fig. 2BC). Nematocyst types were also counted in the mid-body column and proximal region in all polyp types. All three nematocyst types were observed in all polyp types in the proximal portion, although desmonemes dominated the proximal portion of the gastrozooid. The mid-body columns contained a greater percentage of desmonemes and small euryteles in the gastrozooid and large euryteles in the gonozooids and dactylzoooids, though all nematocyst types were present for all polyp types.

### 3.2 *Distribution of fluorescing nematocysts in* Ncol-1::mScar *transgenic*

The nematocyst structural protein minicollagen-1 (Ncol-1), found in all nematocyst types (David et al., 2008), was used to create a nematocyst reporter for *Hydractinia*. Initial injection of ∼80 embryos with *Ncol-1::mScar* plasmid showed variable levels of fluorescence in >70% of planulae within the endoderm and in mature nematocysts across the ectoderm. In the F1 colony, the primary polyp and successive gastrozooids showed a strong signal within the mid-body column of the feeding polyps and scattered throughout the stolonal tissues, as well as in the tentacles and hypostome (mouth region), where mature nematocysts are deployed (Fig. 3). We observed that nearly mature and newly mature nematocysts retained a signal even after migration (as in “super-bright” cell population in Sunagar et al., 2018), and gradually the signal will fade in mature nematocysts (Suppl. Fig. S2). Since nematogenesis is a relatively fast process, occurring in just a few hours (Skaer 1973), it is reasonable to assume that matured mScarlet-I protein will continue to fluoresce in newly matured cells. Since gastrozooids experience a high rate of turnover of nematocysts due to their frequent feeding, this would result in a strong signal in the tentacles. In comparison, a weak signal in the tentacles of gonozooids (not shown) is likely because these cells represent older mature nematocysts, since gonozooids do not participate in active prey-capture and, therefore, experience less turnover. The mid-body columns of gonozooids have highly reduced, if any, signal, which is consistent with nematogenesis occurring elsewhere (such as the stolon) and immature or newly mature nematocysts migrating to the gonozooid as needed. Confocal images confirmed that both immature and mature nematocysts produced an *mScarlet*^+^ signal in gastrozooids (Fig. 3). Additional observations within planulae produced from the F1 animal that express the transgene show a widely distributed signal with a “hotspot” at the proximal portion, where a cluster of mature nematocysts are present (Fig. 3G).

### 3.3 FACS, sequencing, and assembly of nematocyst-enriched transcriptome

Transgenic polyps (∼50 gastrozooids and gonozooids) dissociated into single cells and sorted by FACS, resulted in 252,520 “bright” mScarlet-positive cells and 632,042 mScarlet-negative cells. The resulting samples were considered “live” based on gating for DAPI-negative cells (>98% in both samples). The bright sample included cells that fluoresce at different levels, roughly corresponding with different stages of nematocyst maturation; cells in early development had a strong signal throughout the cell cytoplasm whereas mature nematocysts showed reduced or spotty signaling, which correlates with the reduced expression of *Ncol-1* (Suppl. Fig. S2). While cells in the negative samples did not display any fluorescence or have the morphology of developing nematocysts, some mature nematocysts were present. This is not unexpected given that fluorescence fades in fully matured nematocysts (as noted above). Since mature nematocysts have less transcriptional activity, including toxin expression (Sunagar et al., 2018), this is unlikely to interfere with the results of this study. Light-microscopy images validating the sorted immature and mature nematocysts in the bright sample are shown in Suppl. Fig. S2.

RNA isolation and sequencing for the bright sample produced 107,879,284 reads post-filtering (Q > 30, length >36), which were used to assemble a nematocyst-enriched transcriptome consisting of 96,169 transcripts with a N50 = 2103 bp (Suppl. Table S1). The negative sample produced 131,660,232 post-filtered reads under the same filtered conditions. To validate that the positive sample faithfully represents a nematocyst-enriched dataset, we mapped both the bright and negative reads to the nematocyst-enriched transcriptome using RSEM and obtained transcript per million (TPM) values for relative expression comparisons. Based on the TPM values of nematocyst- or nematogenesis-related genes as well as other types of markers (e.g. neurogenesis, i-cells) (Suppl. Table S2), we demonstrate our transcriptome reliably represents a nematocyst-enriched assembly for *Hydractinia*.

### 3.4 Toxin annotation nematocyst-enriched versus full-tissue

**Fig. 5:**
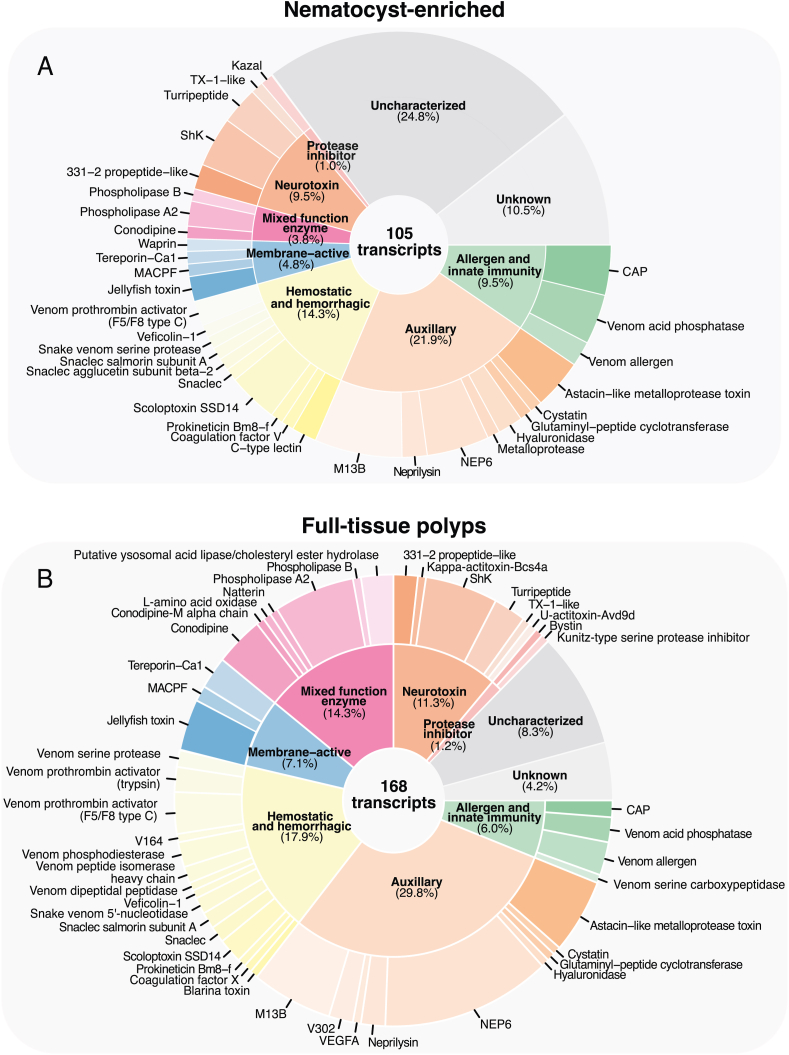
Venom-like gene (VLG) profiles for nematocyst-enriched and full-tissue assemblies. Central number is total VLG transcripts for each assembly. Inner pie charts show the percentage of nine functional categories for total putative VLGs; outer pie chart shows proportion of annotated toxins belonging to each category. Of note, putative venom sequences identified as “cono-“peptides (e.g. conodipine) or other lineage-specific toxins are not truly restricted to these respective groups, thus their presence in a set of cnidarian VLGs is not unexpected.

Putative venom-like genes (VLGs) were determined using a customized annotation pipeline (Fig. 4) for the nematocyst-enriched transcriptome, totaling 105 VLG transcripts (Fig. 5A; Suppl. Table S3). These transcripts were broadly sorted into seven functional categories corresponding to those shown in Klompen et al. (2020), as well as “uncharacterized” (24.8%) and “unknown” (10.5%). Our findings match other cnidarian toxin profiles, including a majority auxiliary (21.9%) and hemostatic and hemorrhagic transcripts (14.3%), as well as neurotoxins (9.5%) and allergen and innate immunity VLGs (9.5%). Membrane-active (4.8%), mixed function enzymes (3.8%), and protease inhibitors (1.0%) are also common amongst cnidarian venoms, and this dataset included several known cnidarian-derived toxins such as MACPF- (Podobnik and Anderluh, 2017) and ShK-containing sequences (Liao et al., 2018), 331–2 propeptides, TX-1-like neuropeptide (Avila Soria, G., 2009) and jellyfish toxins (Brinkman et al., 2015; Klompen et al., 2021). Furthermore, the two jellyfish toxin sequences correspond to those found in Klompen et al. (2021).

In order to compare toxins found in the nematocyst-enriched transcriptome with a whole tissue sample, a similar VLG annotation was conducted for a full-tissue, mixed polyp transcriptome from Sanders and Cartwright (2015), which includes dactylozooid tissues. In total, 168 transcripts were found to be putative toxins, an increase from the number in the nematocyst-enriched dataset (Fig. 5B; Suppl. Table S4). Overall, the broader functional categories for both the whole tissue and nematocyst-enriched dataset are similar, including the dominance of auxiliary and hemostatic and hemorrhagic VLGs and the small number of protease inhibitor sequences. The major categorical differences between the nematocyst-enriched compared and the full-tissue include a reduction in the number of uncharacterized (24.8%–8.3%) and unknown (10.5%–4.2%) transcripts as well as an increase in the number of mixed function enzymes (3.8%–14.3%). Only 43 sequences were highly similar (>98% sequence similarity) between the two datasets (Suppl. Table S5).

### 3.5 Toxins expressed differently between functional polyp types

Using the two different transcriptomic datasets (full-tissue and nematocyst-enriched tissue) we conducted differential expression (DE) analyses to determine; 1) if gastrozooids have a higher level of venom expression than gonozooids, given that gastrozooids experience a higher density of developing nematocysts as observed in the transgenic, and 2) if there are any toxins that show DE between the three different polyp types (Fig. 4). For the nematocyst-enriched dataset, all differentially expressed VLGs are upregulated in gastrozooids (51/51) compared to gonozooids based on the overlap from two DE tests (EBseq and edgeR (Suppl. Table S6). This includes putative toxins from all functional categories, further validating the transgenic observations that nematogenesis (and correspondingly venom production) is more abundant in the gastrozooids than the gonozooids.

For the full-tissue dataset, we used EBseq to analyze DE for the three polyp types, since this test can compare more than two conditions simultaneously. We found 39 differentially expressed genes between gastrozooids, gonozooids, and dactylozooids (Suppl. Fig. S3; Suppl. Table S7). As in the nematocyst-enriched dataset, the majority of VLGs were found to be upregulated in gastrozooids (27/39), consistent with this being an area of active nematogenesis. That said, we found four of the toxins were significantly upregulated in dactylozooids compared to the other polyp types. Of these upregulated toxins, three were membrane-active toxins and the other was a venom peptide isomerase heavy chain peptide (hemostatic and hemorrhagic), all of which may be important in prey-capture. The upregulation of these putative prey-capture toxins in the dactylozooids despite the higher rate of nematogenesis in gastrozooids strongly suggests that particular venoms are being partitioned between functionally distinct polyp types. Additionally, six VLGs are downregulated in gonozooids (i.e. upregulated in gastrozooids and dactylozooid), including four neurotoxins, a Kunitz-type serine protease inhibitor, and unknown sequence, which may be shared prey-capture toxins between the two predatory polyp types. A single unknown VLG is upregulated in gonozooids and one gene similar to conodipine-P1, a phospholipase A2 enzyme from the venom the purple cone snail (*Conus purpurascens* Sowerby I, 1833) (Möller et al., 2019), is downregulated in dactylozooids, which may suggest either a gonozooid-specific or gonozooid-gastrozooid shared VLG or that these two sequences are not true venoms.

## 4 Discussion

### 4.1 Variation in cnidomes across the different polyp types

We found that desmonemes are a dominant nematocyst type in the gastrozooid, representing ∼75% of the nematocysts in the tentacles. Given these nematocysts are thought to only entangle prey and not contain or inject any toxins, this would imply that the 25% of small euryteles in the tentacles contain toxins potent enough to reliably ensure envenomation. These small euryteles are also found at the hypostome (mouth) of the feeding polyps, which may ensure paralysis or lethality of prey before being swallowed by the hydroid. This matches to observed feeding behaviors in these polyps; gastrozooids first ensnare prey in their tentacles and then either bring the paralyzed or dying prey to their mouth while simultaneously bending towards the colony and touching prey with an extended mouth, likely discharging small euryteles directly before consuming them (Suppl. Video S1).

Supplementary data related to this article can be found at https://doi.org/10.1016/j.toxcx.2022.100113.

The following is the supplementary data related to this article:

Multimedia component 4 Multimedia component 4

Although the oral regions of each polyp type have a distinct profile of nematocyst type, the proximal regions of all three polyps contain all nematocyst types (Fig. 2). Nematogenesis has been reported to occur in the mid-body column of the gastrozooid and in the stolon (Kanska and Frank, 2013; Bradshaw et al., 2015). Our transgenic accurately traces areas in the colony of active nematogenesis, given Ncol-1 is initiated during capsule formation early in nematogenesis and mScarlet-I has a relatively short maturation time of 36 min (Bindels et al., 2017). Within gastrozooids, most nematogenesis likely produces desmonemes and small euryteles that migrate from the mid-body to the tentacles, with some portion of the small euryteles migrating to the hypostome. Nematogenesis in the stolonal system (Fig. 3EF) is likely responsible for the production of all nematocyst types. The weak fluorescent signal in the tentacles of the gonozooids and low to no signal in the mid-body column likely reflects low rates of nematocyst turnover, such that a low density of newly matured nematocysts migrate from the stolon through the body column and lose their fluorescent signal at the time of reaching the tentacle. Because the proximal region of the polyps is in close proximity to the stolon system, this results in observations of all nematocyst types as they are migrating throughout the stolon to their respective tissues.

To our knowledge, no other studies have quantified the relative distribution of each nematocyst type across different portions of each type of *Hydractinia* polyp (Fig. 2). Past descriptions primarily focused on the filiform tentacles and hypostome of the gastrozooid and the capitate tentacles of the gonozooids and dactylozooids (e.g. Schuchert et al., 2008). Our cnidome agrees with previous findings that both gonozooids and dactylozooids contain the same types of nematocysts, specifically large euryteles (Mills, 1976).

### 4.2 Venom expression of *Hydractinia*

This study took advantage of our newly established nematocyst-specific reporter line for *Hydractinia* to analyze the expression of VLG sequences specific to the nematocyst cell lineage of this species. Because mature nematocysts have already processed and packaged associated toxins into the closed capsule, venom expression is likely highest during nematocyst development, making this dataset ideal for our study (see Sunagar et al., 2018). Overall, we show that the venom profile of *Hydractinia* is dominated by auxiliary proteins such as metalloproteases and other homeostasis-impairing enzymes (Fig. 5), and includes common cnidarian venom candidates such as phospholipase A2s (Hessinger and Lenoff, 1976; Nevalainen et al., 2004), neurotoxins (Liao et al., 2019), and membrane-active components (Podobnik and Anderluh 2017), such as the cnidarian-specific jellyfish toxins (Klompen et al., 2021).

We compared two different *de novo* assemblies for VLG annotation: the nematocyst-enriched transcriptome constructed in this study (105 VLG transcripts) and a publicly available full-tissue dataset containing a mix of the three different polyp types (168 VLG transcripts) (Sanders and Cartwright 2015) (Fig. 5). Our intention was to disentangle true venoms in the *Hydractinia* nematocysts from potential secondary metabolite toxins used in anti-predation (e.g. Stachowicz and Lindquist, 2000) or toxin-like proteins involved in other functions (von Reumont, 2018). We did observe a reduction in VLGs in the nematocyst-enriched dataset compared to the full-tissue, which could be the result of false positive annotation of non-venom sequences. For example, the proportion of sequences from the mixed function category was significantly lower in the nematocyst-enriched dataset (3.8%) when compared to the full tissue dataset (14.3%), suggesting several of these sequences could be housekeeping enzymes (Fry et al., 2009). However, this reduction could also be explained by the lack of dactylozooid-derived nematocysts in the nematocyst-enriched transcriptome, which yielded some dactylozooid-specific toxins based on DE analysis (Suppl. Table S7). We also observed an increase in uncharacterized VLG sequences in the nematocyst enriched transcriptome (24.8%) compared to the full-tissue (8.3%). This represents a significant increase in the discovery of novel *Hydractinia* VLGs, and is similar to the results from the nematocyst proteome of *Hydra* (Balasubramanian et al., 2012).

While the VLGs from both assemblies were relatively similar in terms of the functional groups, only 43 sequences shared at least 98% sequence similarity between the nematocyst-enriched and full-tissue assemblies (Suppl. Table S5). This may be explained by the different tissue origins; libraries used for mapping and constructing the full-tissue transcriptome were derived from multiple individual colonies in a natural population (Sanders et al., 2014), whereas the animals used to develop the transgenic line are from a long-term lab culture. The different conditions in which these were collected could result in subtle differences in which genes in a gene family are expressed, such that differences between members of a gene family (i.e. paralogs) exceeds the sequence similarity cutoff. That said, these overlapping VLGs, which includes putative peptidases, a phospholipase A2, venom allergen, and MACPF-containing toxin, may represent core venom components in the venoms of *Hydractinia* and make ideal candidates for genomic and functional studies (Suppl. Table S5).

### 4.3 Venom expression across a division of labor

Given the availability of previous polyp-specific data, we attempted to use the full-tissue transcriptome to explore how VLGs are partitioned across the division of labor of *H.* *symbiolongicarus*. For both assemblies, the vast majority of differentially expressed VLGs were upregulated in the gastrozooids (Suppl. Tables S6 and S7), including 9/43 shared VLG sequences, though one was also specifically upregulated in the dactylozooid in the full-tissue dataset (Fig. 4; Suppl. Table S5). In addition to prey-capture and digestion, gastrozooids experience high rates of nematogenesis in their tissues, which would result in the overabundance of putative VLGs being specifically expressed in this polyp type, especially compared to gonozooids where nematogenesis does not occur and there are likely lower rates of nematocyst turnover. This is further emphasized by the lack of upregulated VLGs in gonozooids within both datasets, aside from an unknown sequence in the three-way comparison.

Dactylozooids (predatory polyps) participate in prey-capture but do not consume prey because they lack a mouth, presenting an interesting ecological overlap for comparison with the gastrozooid. It is also assumed that, like in gonozooids, nematogenesis is initiated in the stolons and nematocysts migrate to these polyps. Given that dactylozooids were not present in the nematocyst-enriched analysis, we could only evaluate dactylozooid-specific VLGs in the full-tissue dataset. We found four VLGs were upregulated in dactylozooids (Suppl. Fig. S3), including three hemolytic pore-forming toxins and a venom peptide isomerase heavy chain-like toxin. All of these are potentially important in active predation, in particular the two tereporin-like toxins and jellyfish toxin, both of which are potentially potent pore-forming components. Tereporins are derived from terebrid snails that have close homology to actinoporins in anthozoans (Gorson et al., 2015), suggesting that the sequences found in this dataset could be a novel medusozoa-specific group in this family. Jellyfish toxins are well-known as highly hemolytic and cardiotoxic toxins from medically-relevant box jellyfish (e.g. *Chironex fleckeri*; Brinkman et al., 2015), but have recently been found in a wider variety of medusozoans, including *Hydractinia* (Klompen et al., 2021). Six VLGs were found to be downregulated in gonozooids (i.e. upregulated in prey-capture polyps, dactylozooids and gastrozooids) in the full-tissue comparison, one of which was an unknown gene. The other five included five putative neurotoxins, such as a putative Kunitz-type serine protease inhibitor. Neurotoxins are known to be used by cnidarians for predation and predator deterrence through selectively modifying or blocking ion channels (Turk and Kem 2009), and Kunitz-like toxins are known in cnidarians to possess neurotoxic effects through inhibiting voltage-gated potassium channels (Liao et al., 2018). Each of these putative toxins may aid in incapacitating fast-moving prey, which would be critical to colonies that are otherwise at the whims of the movement of their hermit crab hosts. This patterning shows that prey-capture in particular has an influence on venom partitioning in *Hydractinia*.

Reproductive polyps (gonozooids) and predatory polyps (dactylozooids) contain the same nematocyst types in their tentacles, specifically large euryteles, despite a difference in venom expression within the full-tissue data (Suppl. Fig. S3). This variation can be attributed to either differing rates of nematocyst turnover, given the dactylozooid actively participates in prey-capture while gonozooids are more passively acting in defense of the colony, or potentially more complex regulatory processes during nematogenesis to ensure that both the “right” type of stinging cell and the “right” type of venom are produced. A similar trend was found in the anthozoan *N. vectensis*, where only a subset of similar nematocysts types express NEP3 toxin (Columbus-Shenkar et al., 2018), suggesting that a single nematocyst type can potentially regulate its chemical profiles depending on its trajectory. Regardless, this suggests that venom partitioning in *Hydractinia*, and likely other cnidarians, is managed by regulating different venom components that are distributed to specific tissues. In total, these findings provide convincing evidence that both cnidome and venom composition are partitioned between functionally distinct polyp types in *Hydractinia*, such that this division of labor includes ecologically meaningful venom variation.

## 5 Conclusion

Here, we present a nematocyst-targeted transgenic line for the colonial hydrozoan *Hydractinia symbiolongicarpus* that is a faithful reporter for early nematogenesis through maturation. Using this novel transgenic line, we created a nematocyst-enriched transcriptome to evaluate venom-like genes in *H. symbiolongicarpus*. In combination with our newly generated cnidome profile and existing polyp-specific whole tissue transcriptome, we show that morphologically- and functionally-distinct polyp types of *H. symbiolongicarpus* maintain disparate venom system components. The gastrozooid displays two main nematocyst types while gonozooids and dactylozooids have nearly identical cnidomes dominated by a third type. The majority of upregulated VLGs in feeding polyps are likely due to nematogenesis occurring in these tissues, with reproductive and predatory polyps obtaining nematocysts through migration from the stolon. However, while the reproductive polyps do not display much significant DE in VLGs, dactylozooids contain several significantly upregulated VLGs that likely play important roles in prey-capture. This suggests that regulatory mechanisms ensure that both specific nematocyst types and specific VLGs are localized to the correct tissue. This study showcases the genomic and molecular resources available for *Hydractinia* and its utility for investigating the complex relationship between venom composition and function.

## Author contributions

AMLK: Conceptualization, Methodology, Formal analysis, Data curation, Visualization, Writing – original draft, Writing – review & editing. SMS: Methodology, Writing – review & editing. PC: Conceptualization, Supervision, Funding acquisition, Writing – review & editing.

## Funding

This research was supported by a University of Kansas (KU) Graduate Studies Doctoral Student Research Fund (awarded to AMLK), KU General Research Fund (2105092 awarded to PC) and National Science Foundation (DEB-0953571 awarded to PC). AMLK was supported throughout by a KU Chancellor's Doctoral Fellowship.

## Ethical statement

The animals used in this study do not require NIH Institutional Animal Care and Use Committee (IACUC) approval for research purposes. None of the animals used in this study are endangered species.

## Data availability

Raw reads and nematocyst-enriched *de novo* assembly (TSA: GJUZ00000000.1) from this study are archived in NCBI BioProject PRJNA809365. Raw reads and assembled transcriptome (TSA: GCHW00000000.1) for polyp-specific *Hydractinia* data were obtained from NCBI BioProject PRJNA237004. The *H. symbiolongicarpus* genome is available through the NIH National Human Genome Research Institute (https://research.nhgri.nih.gov/hydractinia/) and corresponding data is archived in the Sequence Read Archive (SRA) under NCBI BioProject PRJNA807936.

## Declaration of competing interest

The authors declare that they have no known competing financial interests or personal relationships that could have appeared to influence the work reported in this paper.

## References

[bib1] Abalde S., Dutertre S., Zardoya R. (2021). A combined transcriptomics and proteomics approach reveals the differences in the predatory and defensive venoms of the molluscivorous cone snail *Cylinder ammiralis* (Caenogastropoda: Conidae). Toxins.

[bib2] Ashwood L.M., Norton R.S., Undheim E.A.B., Hurwood D.A., Prentis P.J. (2020). Characterising functional venom profiles of anthozoans and medusozoans within their ecological context. Mar. Drugs.

[bib3] Ashwood L.M., Mitchell M.L., Madio B., Hurwood D.A., King G.F., Undheim E.A.B., Norton R.S., Prentis P.J. (2021). Tentacle morphological variation coincides with differential expression of toxins in sea anemones. Toxins.

[bib4] Ashwood L.M., Undheim E.A.B., Madio B., Hamilton B.R., Daly M., Hurwood D.A., King G.F., Prentis P.J. (2021). Venoms for all occasions: the functional toxin profiles of different anatomical regions in sea anemones are related to their ecological function. Mol. Ecol..

[bib5] Avila Soria G. (2009). https://eprints.jcu.edu.au/8218.

[bib6] Balasubramanian P.G., Beckmann A., Warnken U., Schnölzer M., Schüler A., Bornberg-Bauer E., Holstein T.W., Özbek S. (2012). Proteome of *Hydra* nematocyst. J. Biol. Chem..

[bib7] Bindels D.S., Haarbosch L., van Weeren L., Postma M., Wiese K.E., Mastop M., Aumonier S., Gotthard G., Royant A., Hink M.A., Gadella T.W.J. (2017). mScarlet: a bright monomeric red fluorescent protein for cellular imaging. Nat. Methods.

[bib8] Boero F., Bouillon J., Piraino S. (2005). The role of Cnidaria in evolution and ecology. Ital. J. Zool..

[bib9] Bradshaw B., Thompson K., Frank U. (2015). Distinct mechanisms underlie oral vs aboral regeneration in the cnidarian *Hydractinia echinata*. Elife.

[bib10] Brinkman D.L., Jia X., Potriquet J., Kumar D., Dash D., Kvaskoff D., Mulvenna J. (2015). Transcriptome and venom proteome of the box jellyfish *Chironex fleckeri*. BMC Genom..

[bib11] Burnett A.L., Sindelar W., Diehl N. (1967). An examination of polymorphism in the hydroid *Hydractinia echinata*. J. Mar. Biol. Ass..

[bib12] Buss, Yund P. (1989). A sibling species group of Hydractinia in the Northeastern United States. J. Mar. Biol. Assoc. U. K..

[bib13] Columbus-Shenkar Y.Y., Sachkova M.Y., Macrander J., Fridrich A., Modepalli V., Reitzel A.M., Sunagar K., Moran Y. (2018). Dynamics of venom composition across a complex life cycle. eLife7.

[bib14] David C.N., Özbek S., Adamczyk P., Meier S., Pauly B., Chapman J., Hwang J.S., Gojobori T., Holstein T.W. (2008). Evolution of complex structures: minicollagens shape the cnidarian nematocyst. Trends Genet..

[bib15] DuBuc T.Q., Schnitzler C.E., Chrysostomou E., McMahon E.T., Febrimarsa Gahan J.M., Buggie T., Gornik S.G., Hanley S., Barreira S.N., Gonzalez P., Baxevanis A.D., Frank U. (2020). Transcription factor AP2 controls cnidarian germ cell induction. Science.

[bib16] Doonan L.B., Lynham S., Quinlan C., Ibiji S.C., Winter C.E., Padilla G., Jaimes-Becerra A., Morandini A.C., Marques A.C., Long P.F. (2019). Venom composition does not vary greatly between different nematocyst types isolated from the primary tentacles of *Olindias sambaquiensis* (Cnidaria: Hydrozoa). Bio. Bull..

[bib17] Dutertre S., Jin A.-H., Vetter I., Hamilton B., Sunagar K., Lavergne V., Dutertre V., Fry B.G., Antunes A., Venter D.J., Alewood P.F., Lewis R.J. (2014). Evolution of separate predation- and defence-evoked venoms in carnivorous cone snails. Nat. Commun..

[bib18] Fautin D.G. (2009). Structural diversity, systematics, and evolution of cnidae. Toxicon.

[bib20] Frank U., Leitz T., Müller W.A. (2001). The hydroid *Hydractinia* : a versatile, informative cnidarian representative: my favorite animal. Bioessays.

[bib21] Frank U., Nicotra M.L., Schnitzler C.E. (2020). The colonial cnidarian Hydractinia. EvoDevo.

[bib22] Fry B.G., Roelants K., Norman J.A. (2009). Tentacles of venom: toxic protein convergence in thehttps://doi.org/10.1038/ncomms4521 Kingdom Animalia. J. Mol. Evol..

[bib23] Gorson J., Ramrattan G., Verdes A., Wright E.M., Kantor Y., Rajaram Srinivasan R., Musunuri R., Packer D., Albano G., Qiu W.-G., Holford M. (2015). Molecular diversity and gene evolution of the venom arsenal of Terebridae predatory marine Snails. Genome Biol. Evol..

[bib24] Hessinger D.A., Lenhoff H.M. (1976). Mechanism of hemolysis induced by nematocyst venom: roles of phospholipase A and direct lytic factor. Arch. Biochem. Biophys..

[bib25] Holstein T. (1981). The morphogenesis of nematocysts in *Hydra* and *Forskalia*: an ultrastructural study. J. Ultrastruct. Res..

[bib26] Huang Y., Niu B., Gao Y., Fu L., Li W. (2010). CD-HIT Suite: a web server for clustering and comparing biological sequences. Bioinformatics.

[bib27] *Hydractinia* Genome Project Portal (2021). https://research.nhgri.nih.gov/hydractinia/.

[bib28] Jungo F., Bougueleret L., Xenarios I., Poux S. (2012). The UniProtKB/Swiss-Prot Tox-Prot program: a central hub of integrated venom protein data. Toxicon.

[bib29] Kanska J., Frank U. (2013). New roles for Nanos in neural cell fate determination revealed by studies in a cnidarian. J. Cell Sci..

[bib30] Kass-Simon G., Scappaticci A.A. (2002). The behavioral and developmental physiology of nematocysts. Can. J. Zool..

[bib31] Klompen A.M.L., Macrander J., Reitzel A.M., Stampar S.N. (2020). Transcriptomic analysis of four cerianthid (Cnidaria, Ceriantharia) venoms. Mar. Drugs.

[bib32] Klompen A.M.L., Kayal E., Collins A.G., Cartwright P. (2021). Phylogenetic and selection analysis of an expanded family of putatively pore-forming jellyfish toxins (Cnidaria: medusozoa). Gen. Biol. Evol..

[bib33] Künzel T., Heiermann R., Frank U., Müller W., Tilmann W., Bause M., Nonn A., Helling M., Schwarz R.S., Plickert G. (2010). Migration and differentiation potential of stem cells in the cnidarian *Hydractinia* analysed in eGFP-transgenic animals and chimeras. Dev. Biol..

[bib34] Lange R., Plickert G., Müller W.A. (1989). Histoincompatibility in a low invertebrate, *Hydractinia echinata*: analysis of the mechanism of rejection. J. Exp. Zool..

[bib35] Leng N., Dawson J.A., Thomson J.A., Ruotti V., Rissman A.I., Smits B.M.G., Haag J.D., Gould M.N., Stewart R.M., Kendziorski C. (2013). EBSeq: an empirical Bayes hierarchical model for inference in RNA-seq experiments. Bioinformatics.

[bib36] Lewis Ames C., Macrander J. (2016). Evidence for an alternative mechanism of toxin production in the box jellyfish *Alatina alata*. Integr. Comp. Biol..

[bib37] Li B., Dewey C.N. (2011). RSEM: accurate transcript quantification from RNA-Seq data with or without a reference genome. BMC Bioinf..

[bib38] Liao Q., Li S., Siu S.W.I., Yang B., Huang C., Chan J.Y.-W., Morlighem J.-É.R.L., Wong C.T.T., Rádis-Baptista G., Lee S.M.-Y. (2018). Novel Kunitz-like peptides discovered in the zoanthid *Palythoa caribaeorum* through transcriptome sequencing. J. Proteome Res..

[bib39] Liao Q., Feng Y., Yang B., Lee S.M.-Y. (2019). Cnidarian peptide neurotoxins: a new source of various ion channel modulators or blockers against central nervous systems disease. Drug Discov. Today.

[bib40] Macrander J., Broe M., Daly M. (2016). Tissue-specific venom composition and differential gene expression in sea anemones. Genome Biol. Evol..

[bib41] Macrander J., Brugler M.R., Daly M. (2015). A RNA-seq approach to identify putative toxins from acrorhagi in aggressive and non-aggressive *Anthopleura elegantissima* polyps. BMC Genom..

[bib42] McClounan S., Seymour J. (2012). Venom and cnidome ontogeny of the cubomedusae *Chironex fleckeri*. Toxicon.

[bib44] Mills C.E. (1976). *Podocoryne selena*, a new species of hydroid from the Gulf of Mexico, and a comparison with *Hydractinia echinata*. Biol. Bull..

[bib45] Mistry J., Chuguransky S., Williams L., Qureshi M., Salazar G.A., Sonnhammer E.L.L., Tosatto S.C.E., Paladin L., Raj S., Richardson L.J., Finn R.D., Bateman A. (2021). Pfam: the protein families database in 2021. Nucleic Acids Res..

[bib46] Möller C., Davis W.C., Clark E., DeCaprio A., Marí F. (2019). Conodipine-P1-3, the first Phospholipases A2 characterized from injected cone snail venom. Mol. Cell. Proteomics.

[bib47] Morgenstern D., King G.F. (2013). The venom optimization hypothesis revisited. Toxicon.

[bib48] Munro C., Zapata F., Howison M., Siebert S., Dunn C.W. (2021).

[bib49] Nevalainen T.J., Peuravuori H.J., Quinn R.J., Llewellyn L.E., Benzie J.A.H., Fenner P.J., Winkel K.D. (2004). Phospholipase A2 in Cnidaria. Comp. Biochem. Physiol. B Biochem. Mol. Biol..

[bib50] Östman C. (2000). A guideline to nematocyst nomenclature and classification, and some notes on the systematic value of nematocysts. Sci. Mar..

[bib51] Podobnik M., Anderluh G. (2017). Pore-forming toxins in Cnidaria. Semin. Cell Dev. Biol..

[bib52] Quiroga-Artigas G., Duscher A., Lundquist K., Waletich J., Schnitzler C.E. (2020). Gene knockdown via electroporation of short hairpin RNAs in embryos of the marine hydroid *Hydractinia symbiolongicarpus*. Sci. Rep..

[bib53] R Core Team (2021). https://www.R-project.org/.

[bib54] RStudio Team (2021). https://www.rstudio.com/.

[bib55] Rachamim T., Sher D. (2012). What *Hydra* can teach us about chemical ecology how a simple, soft organism survives in a hostile aqueous environment. Int. J. Dev. Biol..

[bib56] Robinson M.D., McCarthy D.J., Smyth G.K. (2010). edgeR: a Bioconductor package for differential expression analysis of digital gene expression data. Bioinformatics.

[bib57] Sachkova M.Y., Macrander J., Surm J.M., Aharoni R., Menard-Harvey S.S., Klock A., Leach W.B., Reitzel A.M., Moran Y. (2020). Some like it hot: population-specific adaptations in venom production to abiotic stressors in a widely distributed cnidarian. BMC Biol..

[bib58] Sanders S.M., Shcheglovitova M., Cartwright P. (2014). Differential gene expression between functionally specialized polyps of the colonial hydrozoan *Hydractinia symbiolongicarpus* (Phylum Cnidaria). BMC Genom..

[bib59] Sanders S.M., Cartwright P. (2015). Interspecific differential expression analysis of rna-seq data yields insight into life cycle variation in hydractiniid hydrozoans. Genom. Biol. Evol..

[bib60] Sanders S.M., Ma Z., Hughes J.M., Riscoe B.M., Gibson G.A., Watson A.M., Flici H., Frank U., Schnitzler C.E., Baxevanis A.D., Nicotra M.L. (2018). CRISPR/Cas9-mediated gene knockin in the hydroid *Hydractinia symbiolongicarpus*. BMC Genom..

[bib61] Schendel V., Rash L.D., Jenner R.A., Undheim E.A.B. (2019). The diversity of venom: the importance of behavior and venom system morphology in understanding its ecology and evolution. Toxins.

[bib62] Schuchert P. (2008). The European athecate hydroids and their medusae (Hydrozoa, Cuidaria): Filifera Part 3. Rev. Suisse Zool..

[bib63] Schuchert P. (2014). Observations on *Hydractinia aculeata* (Hydrozoa, Cnidaria). Rev. Suisse Zool..

[bib64] Sher D., Zlotkin E. (2009). A hydra with many heads: protein and polypeptide toxins from *Hydra* and their biological roles. Toxicon.

[bib65] Skaer R.J. (1973). The secretion and development of nematocysts in a siphonophore. J. Cell Sci..

[bib66] Stachowicz J.J., Lindquist N. (2000). Hydroid defenses against predators: the importance of secondary metabolites versus nematocysts. Oecologia.

[bib67] Sunagar K., Columbus-Shenkar Y.Y., Fridrich A., Gutkovich N., Aharoni R., Moran Y. (2018). Cell type-specific expression profiling unravels the development and evolution of stinging cells in sea anemone. BMC Biol..

[bib68] Surm J.M., Smith H.L., Madio B., Undheim E.A.B., King G.F., Hamilton B.R., van der Burg C.A., Pavasovic A., Prentis P.J. (2019). A process of convergent amplification and tissue-specific expression dominates the evolution of toxin and toxin-like genes in sea anemones. Mol. Ecol..

[bib69] Turk T., Kem W.R. (2009). The phylum Cnidaria and investigations of its toxins and venoms until 1990. Toxicon.

[bib70] Underwood A.H., Seymour J.E. (2007). Venom ontogeny, diet and morphology in *Carukia barnesi*, a species of Australian box jellyfish that causes Irukandji syndrome. Toxicon.

[bib71] Verdes A., Simpson D., Holford M. (2018). Are fireworms venomous? Evidence for the convergent evolution of toxin homologs in three species of fireworms (Annelida, Amphinomidae). Genom. Biol. Evol..

[bib72] von Reumont B.M., Campbell L.I., Richter S., Hering L., Sykes D., Hetmank J., Jenner R.A., Bleidorn C. (2014). A polychaete's powerful punch: venom gland transcriptomics of *Glycera* reveals a complex cocktail of toxin homologs. Genom. Biol. Evol..

[bib73] von Reumont B.M. (2018). Studying smaller and neglected organisms in modern evolutionary venomics implementing RNASeq (transcriptomics)—a critical guide. Toxins.

[bib74] Walker A.A., Mayhew M.L., Jin J., Herzig V., Undheim E.A.B., Sombke A., Fry B.G., Meritt D.J., King G.F. (2018). The assassin bug *Pristhesancus plagipennis* produces two distinct venoms in separate gland lumens. Nat. Commun..

[bib75] Weill R. (1934). Contribution a l'etude des Cni- daires et de leurs n6matocystes. Trav. Stat. Zool. d. Wimereux.

