# Supplementary material for: Venom system variation and the division of labor in the colonial hydrozoan Hydractinia symbiolongicarpus
Source: Toxicon X. 2022 Mar 4;14:100113. doi: 10.1016/j.toxcx.2022.100113 (PMC8917316; doi:10.1016/j.toxcx.2022.100113)
Supplement: Multimedia component 1 [file mmc1.docx]

**SUPPLEMENTAL MATERIALS**

**Captions for:**

**Supplemental Table**

**Supplemental Video**

**Supplemental File**

**Supplemental Figures**

**Supplemental References**

**Supplemental Table S1-7: Multi-sheet excel file for data produced in this study.** Sheet 1: Assembly statistics for nematocyst-enriched transcriptome. N10, N50, median length and average length based on all transcript contigs. Sheet 2: Relative expression of nematocyst marker genes and others cell type markers based on TPM values from bright and negative reads mapped to nematocyst-enriched assembly. Yellow highlight indicates the *minicollagen-1* gene targeted in this study and *mScarlet-I* inserted via transgenesis. Sheet 3: Nematocyst-enriched VLG annotation. Sheet 4: Full-tissue VLG annotation. Sheet 5: Putative VLGs that overlap (98% sequence similarity) between the two assemblies. Genes in bold are those that have significant differential expression results from both datasets. Sheet 6: Differential expression results for nematocyst-enriched VLGs, which compared gonozooids with gastrozooids. Sheet 7: Differential expression results for full-tissue VLGs, with compared all three polyp types.

**Supplemental Video S1: *Hydractinia* feeding behavior.** Gastrozooids entangle 1-day old brine shrimp nauplii (*Artemia* sp.) in their tentacles followed by a bending of the gastrozooid inwards, towards the colony. The gastrozooid extends the mouth and briefly touches the shrimp, which appears to either cause full paralysis or lethality. The polyp then expands its mouth to consume the brine shrimp (not shown). Other surrounding gastrozooids may also react to a neighboring polyp by bending inwards towards the prey item.

**Supplemental File S1: Sequence file for *Ncol-1::mScar* donor plasmid.**

TTGTTGCTATCGACACATCAGAAATGTTCCTATTATTTTTATTAGTCAGGAACATGCTGAACTGTTTATCAAAATTTTAAGAAATTTTTATAATATTTTAAGACTAATATTGGGGCTCGCTTTTATGAGATTATTTATCTGTTCTTTTTAAAATAAGAAACATCTTACACAGTCATTTTGTAATGTATCTCCTTTCAAAAAACAGTTAGCAGTTTTGTGTTTCCGAAAGTTTTATTAAGAAATAATGCGTAACTAGATCGATACAAAATTATTATAGCAGATTGATTGCATCCATCAGCCTGTCCGAAATTATCTCACAGCAATTTGGTGCGCTATTGTAAAGCTGAAAGTCGAAATTAACGTTATTTATTTTGTTACAGCCTGGTTTTATGAATTTTATTCACTTGTTATATTTTTTATCTTTTATTTTTAAAAACAATTATTATAAAATAGAAAGCGTAGCACTTTTGAAAAGAAAGGAACGCGGGTGTAAAAACAACAACAAATAGTGTTGTGATTTTGACTGTGTGTAAAATGCTCTATTTTCAAAAAAACAGACGTACCTTCTCTTCGTTTGCGAGTGGAGACGTCTAGATAAAGATTTTATTTAACTTCGACGGGAACTAGATCCAAGCTCATCCTTTCTCATGCAAATAGACAGAAAAATTTTAAAATTTAAATTTGTTTTACTGTTTATCGCCTCTCCCAAAAAAAAAAAAAAAAAGGAATTTAAAAATATAGATATTTGAAATTCTCTTAACCTCAAAACTTTTTTAAAAATCAAAAATATATATTATTCGTTGAAATCTGTGCAAAAAAAGTGTCCAAACTTTTAAAACTCGTCAAAAGTAAAAACGTTTAAATTTTTTTCACCTAGAACGATTTTATGCGCAATAATAAATTCACTGCGTTCAAACGATCGCGGAAGAAATCGAAGCTCGTGTCAAATGGAATGCTGCACAAAATAGTACCATCTATTGAGACACTAGTAAATTTTCGAATGCTTCGTTACTCTCACAATTTTTTACAGGGGGTAAAAAAACAGAGAATTTAGTATGAAAACAGAACAAAAATACGACGAAATAACTATTTCTTATGGAAAGTGTGTAAATTATTTTGAAAATGAAGTTTGAAAATTCAAATGTGGTTTAAAAAAATTAGTTAGGTGCAGAATAAAACTATATCTAACATTTTTGTACCCCCTGATAAAATTGATATGTTTTTAATTTGACAACTGAATCGTAAAATTGTTCGAAGTTGAGGATTAAATGATCATTCAGCATGAGGCTACGCTACAGCGCGCATGCCTATTACCCGGTGAGAAATGTCATGCTTGGGTAACCCTGTGTGCGCATGATCAATTACATTACAAAATCAACCAATCAAATTACAGAAAATCATAAAATCTTGAGCAGCTTACTTCTGTGCAGGTATAAAGAAAGTTATTTCGAAAAGTCTAATTATCATTCATACCTCAATTGCCATTAGAATTGGACGGAGTGCGGAAAAGGGTAAGGTAATTATTTTTTATATAAACTGTTTCTTGTTGTCTTCTTCTTATTATTGTTTATCATTGATATATTGTTTAATATTGTTACATACTATCGTATTTATATTTGTGTATTTCGAGTAATTACAACAACAAAATTTATTTTATTTGCTTTTCGCCTTCACAATCATATTTTAAATTAAAATTATTTTTATGAGACCAAAAAATGTTTTTTTCTTATGCTCTATCTTACAAATTCTCCTTGGTACTTCTCAGCGTGACTAATACACATTTATACTATTTAAGACCTACACAACTAAATAAACAAAAATAAAGATTTTAACTAAAACAATATTTTAGGAAAAAAAATATACAATACAAATATACAATCCACCTCCTAAATTAAATCAATAATCTGTTAGCACAAATAAAATATAAAATACGTTTTCGATTTTAGCGATTATAACAATCCTACAGTAATGGTATCTAAAGGTGAAGCAGTTATAAAAGAGTTTATGAGATTTAAAGTTCATATGGAAGGTTCCATGAATGGACATGAATTTGAAATTGAAGGAGAAGGTGAAGGTAGACCATACGAAGGAACACAAACAGCTAAGTTAAAAGTTACAAAAGGAGGTCCACTGCCATTCTCGTGGGATATTTTATCTCCTCAATTTATGTATGGTTCCAGAGCTTTCATCAAGCACCCAGCTGATATACCGGACTATTATAAACAAAGTTTTCCGGAGGGATTCAAATGGGAAAGAGTTATGAACTTTGAAGACGGAGGAGCTGTTACAGTAACCCAGGATACCAGTCTGGAAGATGGTACTTTGATTTACAAAGTTAAATTACGAGGAACAAATTTTCCGCCTGATGGCCCTGTAATGCAGAAGAAGACAATGGGTTGGGAGGCATCGACTGAGCGTTTATACCCCGAAGACGGCGTCTTGAAGGGAGATATAAAAATGGCTTTACGATTAAAGGACGGTGGCCGTTATCTAGCTGATTTTAAAACGACATATAAGGCAAAGAAACCCGTTCAAATGCCTGGTGCTTATAATGTTGATCGGAAATTAGAGATCACATCACACAACGAAGATTATACAGTTGTCGAACAATATGAGCGAAGTGAAGGCAGGCACAGTACTGGAGGAATGGATGAACTATACTAAATGATGACAAGGAATTAGATATTTTTCAAATTACTTCTTTTCTTGAACTGATACTTTTCATATGGTTATTGACACAGACGCGAACAGATTAGCATCTACTCGTCCAAGATAGATTGAATGGATTGGAATATATATTATATTGTTAAATTATAGTTTCAATTGTGTGGGACTTTCCATGCAACCCGGAATCTCATTATTTTTTTTTTGAAAGATATATGTGAATTTTAATGCATGATCATATGAAAGAATAAATATTTTTCTATCCATAAAATTTTGATCTTTTTTTCTTTCTTATCAAAAGTAATCTACAAGAATTGGACTAACTTGTTTTTGAATGCATTTAATTTTATTTCCAAAAAGATTTACAAAATATCCAACGCTTCAAAAGTATATAATTTTTCTACAAATAACATTTTTTCTAAAATTAACTACAAACAATTATATATCCCTTGAGATTTAAACTTCCGAGGTTTAAAAATAACCTTGCAGTACTCCATGATTATGGTATTTTTTACAGAAAATTAAGCCTAATAGATCAAAAATTATGAACACACTCATGGCCTAGCTCTAATAAATGCTAGAAAAGCAAGTTAAACATCTGCTCTAACAATTTTGGCACCCAGTTATCGGGAAAAAAATAAAAATCAAGCCAGCAAATCGCTCTACATATCTCAGTTTTCTTGTTGTAACCATATGTTGTAGAAATCTTCGATAACTCAACGCGTCATTTCTGCATGATCTTTATAAGTTGAAGCTTGAAAAAATATGCCTAACTAAATCGTTTGTTTACTGTCAGGAAATAAAATTTTGTGGCAATGTCCAAAGATGGCGTTTAACATTACCTAGACTAACATTTGAGTTCTTTTGAATTAAAAACACCAGAAATACATTCAGTCGAAGGTTTTCTTTGATCAAAATTTCGAAGGTTGTATAGAAGGCAGAGAAAGGGTTTGACAGATTCGCTATTTCTTGTTTTGTAAAAGGTATAAGCGTTGCAGTAACTACTACGAAAACACGCATCGAAGCATTGACTCACTGTATCAACGTGAGTATAATTTTTGGTTATGATAGCTAGGTTAACAAATTTCTTGTTATACAGGAACCCGTCCTTATAGACAGCATTCTGGCAATGTTCTCCAAAAATTGCGTCACTTGTTAACAATATCCAGTGAATACATAGATAAAACAATGCGTGATTTAATTGGATGAACATTTTATTAATCAATCGGTTTTCTTGTCAGATTTTCTTTGTTAAAAAACGAGTTACCTTGAGTGTTTCTTGATCACCTTAATTTTCCTTTTAATTCTTCTTTTGTTTAAAGCATTTTGATTTTTCCAAACATCTGCAAGTACCCCTACTTAACTTTTCAATCGTAAACTAACAAAACTTACTTCTATTGTGTATGTGTGAAGTCTACTCATAAACTTG

*Ncol-1* 5’ genomic region (1968 bp)

mScarlet-I coding region (696 bp)

*Ncol-1* 3’ genomic region (1429 bp)

**Supplemental Figure S1: Results from FACS of dissociated *Ncol-1::mScar* *Hydractinia*.** A)
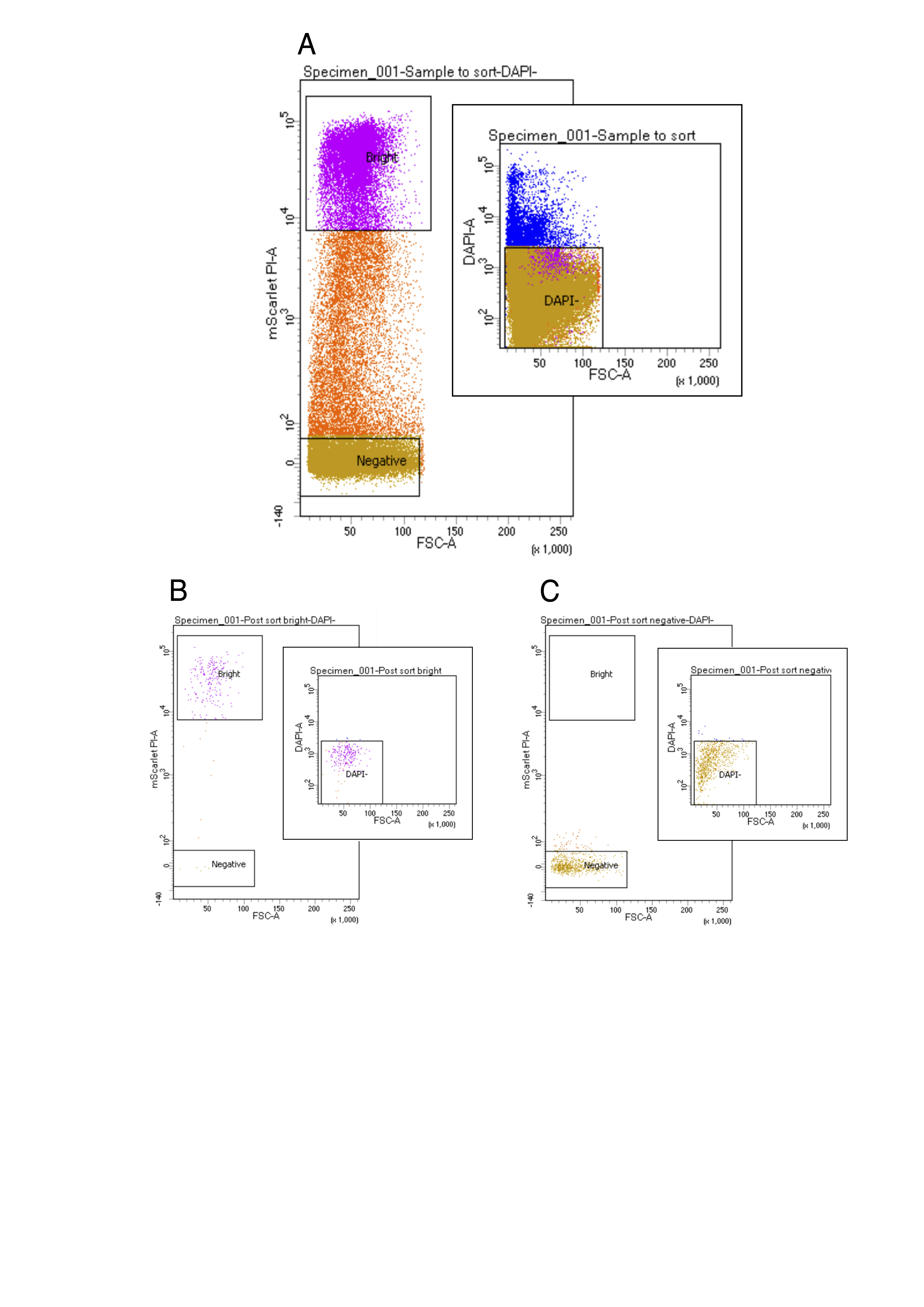
 Total results from sorting experiment, showing the gating for positive and negative cell samples. Insert shows gating for live sorting via DAPI-negative cells. B) Post-sort analysis for positive “bright” sample. C) Post-sort analysis for negative sample.

**Supplemental Figure S2: Post-sort visualization of positive “bright” cells from FACS.** A)
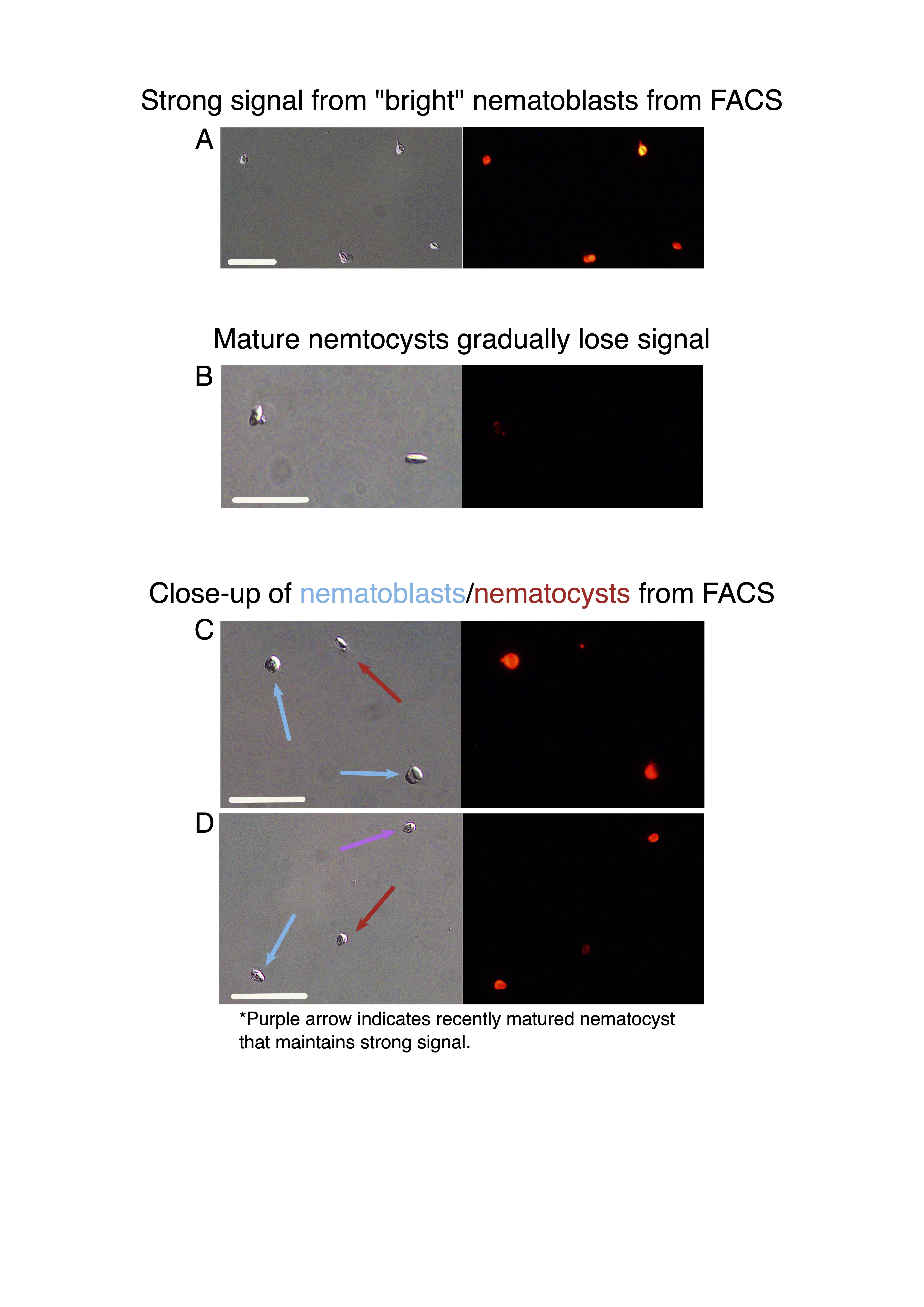
 Immature nematocysts (nematoblasts) show the strongest fluorescence signal. B) Fluorescence in mature nematocysts gradually fades, such that some mature cells sort into both the bright and negative samples (not shown). C-D) Mix of immature (blue) and mature (red) nematocysts and the resulting variability in signal. Purple (D) arrow shows a nearly mature/mature desmoneme that retains a strong signal. Imaged using Lumenera Infinity 3 camera with Infinity Analyze v7 software. Scale bar = 20 μm.

**
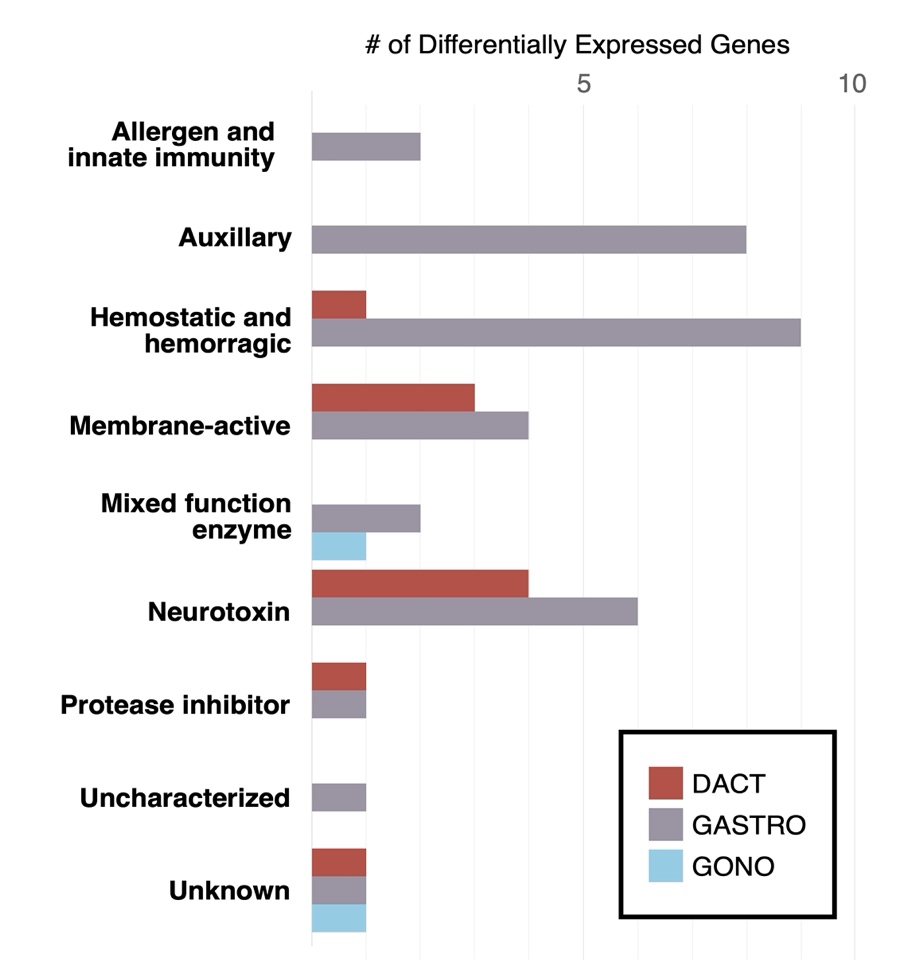
**

**Supplemental Figure S3: Upregulated differentially expressed VLGs from three-polyp comparison using EBSeq.** A gene found to be downregulated in one polyp type was considered upregulated in the other two types (e.g. unknown VLG downregulated in gonozooid = +1 for gastrozooid and dactylozooid). Gene annotations are shown in Supplemental Table S7.

**Supplemental References**

Engel, U., Oezbek, S., Engel, R., Petri, B., Lottspeich, F., Holstein, T.W., 2002. Nowa, a novel protein with minicollagen Cys-rich domains, is involved in nematocyst formation in *Hydra*. Journal of Cell Science 115, 3923–3934. <https://doi.org/10.1242/jcs.00084>

Fedders, H., Augustin, R., Bosch, T.C.G., 2004. A Dickkopf- 3-related gene is expressed in differentiating nematocytes in the basal metazoan Hydra. Dev Genes Evol 214, 72–80. <https://doi.org/10.1007/s00427-003-0378-9>

Flici, H., Schnitzler, C.E., Millane, R.C., Govinden, G., Houlihan, A., Boomkamp, S.D., Shen, S., Baxevanis, A.D., Frank, U., 2017. An evolutionarily conserved Soxb-Hdac2 crosstalk regulates neurogenesis in a cnidarian. Cell Reports 18, 1395–1409. <https://doi.org/10.1016/j.celrep.2017.01.019>

Gahan, J.M., Schnitzler, C.E., DuBuc, T.Q., Doonan, L.B., Kanska, J., Gornik, S.G., Barreira, S., Thompson, K., Schiffer, P., Baxevanis, A.D., Frank, U., 2017. Functional studies on the role of Notch signaling in *Hydractinia* development. Developmental Biology 428, 224–231. <https://doi.org/10.1016/j.ydbio.2017.06.006>

Hwang, J.S., Takaku, Y., Chapman, J., Ikeo, K., David, C.N., Gojobori, T., 2008. Cilium evolution: Identification of a novel protein, nematocilin, in the mechanosensory cilium of *Hydra* nematocytes. Mol. Biol. Evol. 25, 2009–2017. <https://doi.org/10.1093/molbev/msn154>

Hwang, J.S., Takaku, Y., Momose, T., Adamczyk, P., Ozbek, S., Ikeo, K., Khalturin, K., Hemmrich, G., Bosch, T.C.G., Holstein, T.W., David, C.N., Gojobori, T., 2010. Nematogalectin, a nematocyst protein with GlyXY and galectin domains, demonstrates nematocyte-specific alternative splicing in Hydra. Proc. Natl. Acad. Sci. U.S.A. 107, 18539–18544. <https://doi.org/10.1073/pnas.1003256107>

Kanska, J., Frank, U., 2013. New roles for Nanos in neural cell fate determination revealed by studies in a cnidarian. J. Cell Sci. 126, 3192–3203. <https://doi.org/10.1242/jcs.127233>

Katsukura, Y., Ando, H., David, C. N., Grimmelikhuijzen, C. J., Sugiyama, T. 2004. Control of planula migration by LWamide and RFamide neuropeptides in *Hydractinia echinata*. J. Exp. Biol., 207, 1803-1810. <https://doi.org/10.1242/jeb.00974>

Koch, A.W., Holstein, T.W., Mala, C., Kurz, E., Engel, J., David, C.N., 1998. Spinalin, a new glycine- and histidine-rich protein in spines of Hydra nematocysts. J. Cell Sci. 111, 1545–1554. <https://doi.org/10.1242/jcs.111.11.1545>
