# Supplementary figures and images for: Venom system variation and the division of labor in the colonial hydrozoan Hydractinia symbiolongicarpus
Source: Toxicon X. 2022 Mar 4;14:100113. doi: 10.1016/j.toxcx.2022.100113 (PMC8917316; doi:10.1016/j.toxcx.2022.100113)

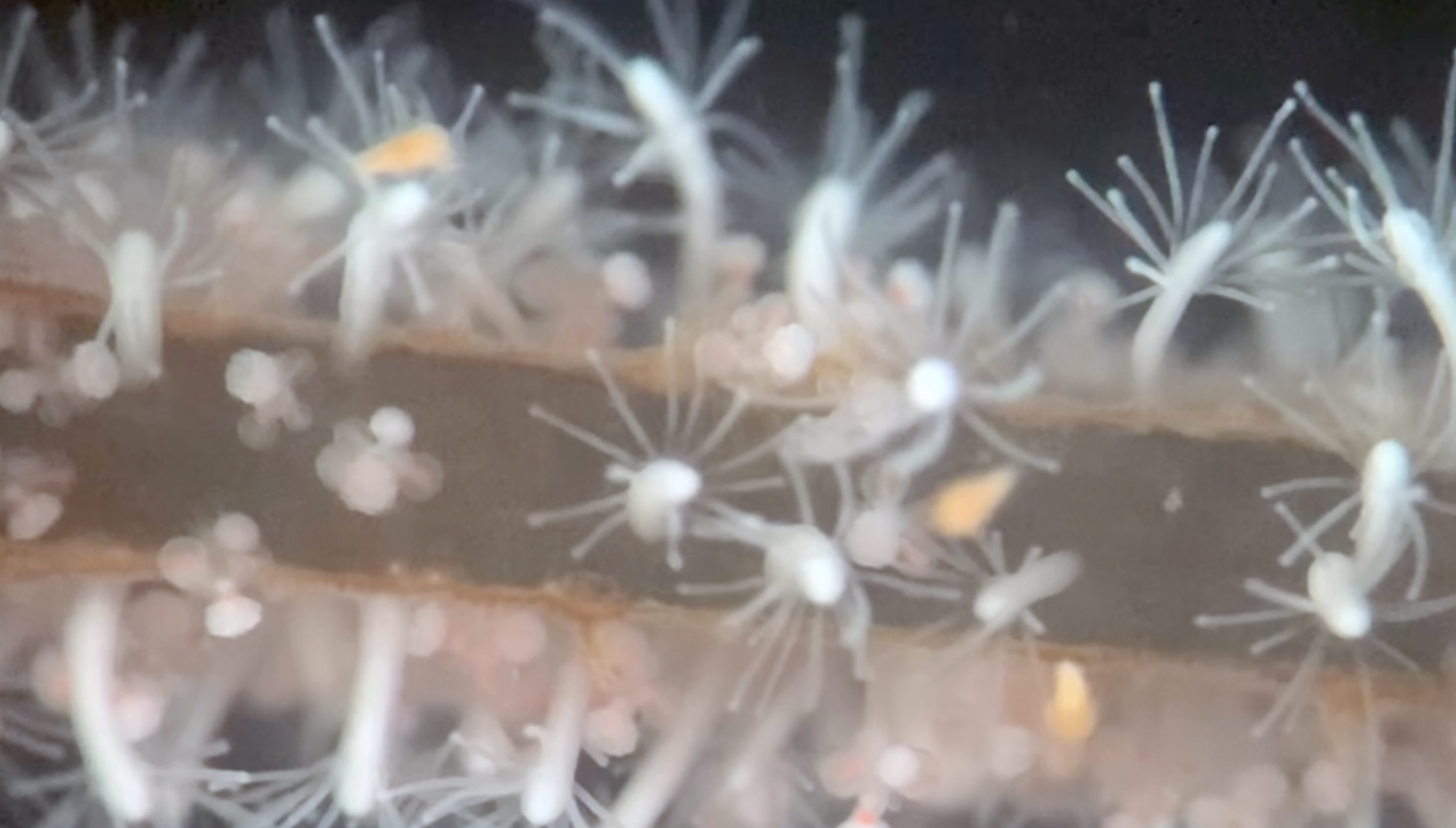

Feeding behavior of  
*Hydractinia* gastrozooids

Supplement: Multimedia component 3 [file mmc3.pdf]
